# Protective Effects of Ferulic Acid against Chronic Cerebral Hypoperfusion-Induced Swallowing Dysfunction in Rats

**DOI:** 10.3390/ijms18030550

**Published:** 2017-03-03

**Authors:** Takashi Asano, Hirokazu Matsuzaki, Naohiro Iwata, Meiyan Xuan, Shinya Kamiuchi, Yasuhide Hibino, Takeshi Sakamoto, Mari Okazaki

**Affiliations:** 1 Laboratory of Pharmacology, Faculty of Pharmaceutical Sciences, Josai University, Saitama 350-0295, Japan; gyd1401@josai.ac.jp (T.A.); ma-tsu@josai.ac.jp (H.M.); 2 Laboratory of Immunobiochemistry, Faculty of Pharmaceutical Sciences, Josai University, Saitama 350-0295, Japan; n-iwata@josai.ac.jp (N.I.); kamiuchi@josai.ac.jp (S.K.); seitaib@josai.ac.jp (Y.H.); 3 Laboratory of Organic and Medicinal Chemistry, Faculty of Pharmaceutical Sciences, Josai University, Saitama 350-0295, Japan; genbien@josai.ac.jp (M.X.); sakamoto@josai.ac.jp (T.S.)

**Keywords:** ferulic acid, cerebral hypoperfusion, swallowing dysfunction, oxidative stress, dopaminergic neuron, substance P

## Abstract

Ferulic acid (FA), a phenolic phytochemical, has been reported to exert antioxidative and neuroprotective effects. In this study, we investigated the protective effects of FA against the dysfunction of the swallowing reflex induced by ligation of bilateral common carotid arteries (2VO) in rats. In 2VO rats, topical administration of water or citric acid to the pharyngolaryngeal region evoked a diminished number of swallowing events with prolonged latency compared to sham-operated control rats. 2VO rats had an increased level of superoxide anion radical, and decreased dopamine and tyrosine hydroxylase enzyme levels in the striatum, suggesting that 2VO augmented cerebral oxidative stress and impaired the striatal dopaminergic system. Furthermore, substance P (SP) expression in the laryngopharyngeal mucosa, which is believed to be positively regulated by dopaminergic signaling in the basal ganglia, was decreased in 2VO rats. Oral treatment with FA (30 mg/kg) for 3 weeks (from one week before 2VO to two weeks after) improved the swallowing reflex and maintained levels of striatal dopamine and laryngopharyngeal SP expression in 2VO rats. These results suggest that FA maintains the swallowing reflex by protecting the dopamine-SP system against ischemia-induced oxidative damage in 2VO rats.

## 1. Introduction

Cerebrovascular disease including stroke is a significant cause of morbidity and mortality, and is associated with various complications resulting in life-long disabilities [1]. Oropharyngeal dysphagia attributed to impaired swallowing reflex in the upper aerodigestive tract is a common complication in post-stroke patients, which increases the risk of lethal aspiration pneumonia [2,3]. Despite the high incidence of oropharyngeal dysphagia among patients with stroke, fundamental and effective pharmacotherapeutics have not been well-established yet. New efficacious approaches including alternative therapy for prevention and treatment of post-stroke swallowing dysfunction are needed for better preservation of the quality of life and prognosis in the patients.

Swallowing is a complex physiological process that can be initiated either voluntarily or reflexively, whereas the pharyngeal phase is a stereotyped movement of the tongue and laryngopharyngeal structures. Reflexive swallowing is directly evoked by the activity of the swallowing central pattern generator (CPG) located in the medulla oblongata, which is activated by sensory inputs of afferent fibers (i.e., the superior laryngeal nerve and the glossopharyngeal nerve) distributed on the laryngopharyngeal mucosa responding to mechanical and chemical stimulation [4]. Patients with acute basal ganglia infarcts post-stroke frequently exhibit impairment of the swallowing reflex as well as an attenuated cough reflex leading to silent aspiration during sleep, which may be due to the diminution of sensory inputs to the CPG. In fact, the nigrostriatal extrapyramidal nervous system plays a significant role in maintaining the sensitivity of the trigger zone for initiating involuntary reflex swallowing in the laryngopharyngeal region, and is of particular interest as a therapeutic target for oropharyngeal dysphagia because of its pathological sensitivity [5]. Dopamine in the nigrostriatal system is believed to elevate levels of substance P (SP), an essential trigger mediator for the swallowing reflex, in sensory fibers of the laryngopharyngeal region [6,7,8]. Basal ganglia infarction frequently impairs the dopaminergic neurotransmission and leads to a decrease in SP in the region [9,10]. It has been suggested that the impairment of dopaminergic transmission also relates to oropharyngeal dysphagia associated with Parkinson’s disease and aging [11,12]. Thus, the dysfunction of dopaminergic neurons in the basal ganglia with subsequent reduction of laryngopharyngeal SP seems to be involved in oropharyngeal dysphagia, although it is likely that the degeneration in the brainstem and other forebrain areas, as well as attenuation in cortico-striatal excitability, contributes to a more severe and complicated clinical pathological condition. These observations suggest that treatment with drugs that can protect the nigrostriatal dopaminergic system and/or enhance the dopaminergic transmission prevents post-stroke oropharyngeal dysphagia. Indeed, therapeutic drugs that can increase dopaminergic neurotransmission, such as levodopa [13], amantadine [14], and some dopamine receptor agonists [15], have been shown to improve the swallowing reflex. Cilostazol, which is a potent type III phosphodiesterase inhibitor frequently used for secondary stroke prevention, has been reported to decrease the prevalence of aspiration pneumonia in stroke patients [16,17]. Basic studies using a rat cerebral hypoperfusion model suggest that the antiaspiration effect of cilostazol is ascribed in part to the improvement of the swallowing reflex bestowed by its cerebroprotective property enhancing the cyclic AMP-responsive element binding protein (CREB) pathway [8,18]. However, clinical use of these synthetic compounds has been severely restricted due to side-effects, such as movement disorders or bleeding tendency, associated with long-term use.

Ferulic acid (4-hydroxy-3-methoxycinnamic acid, FA) is one of the several phenolic phytochemical compounds found in various plants such as rice bran and coffee beans, and shows a broad spectrum of biological and pharmacological properties including anti-inflammatory, antihypertensive [19], antidiabetic [20,21], and anticancer [22] effects. FA exerts a strong cytoprotective activity due to both the ability to scavenge free radicals [23,24] and activate cellular responses against oxidative stress [25,26]. FA has gained considerable attention as a low-toxicity dietary supplement for its potential against cognitive symptoms in dementia [26,27]. In addition, several studies have reported that the treatment with FA shows significant neuroprotective effects against cerebral ischemic injury by attenuating oxidative stress and inflammation in rodent models [28,29]. Based on these observations, we assumed that chronic treatment with FA can protect the nigrostriatal system against ischemia-induced oxidative damage and prevent impairment of swallowing reflex. In this study, to investigate whether FA can be an effective prophylactic against oropharyngeal dysphagia, we examined the effects of FA using a rat model with swallowing dysfunction induced by chronic cerebral hypoperfusion associated with a ligation of bilateral common carotid arteries (2VO). We also estimated the protective effect of FA on striatal dopaminergic activity by histochemically determining neuronal oxidative stress and apoptosis as well as tyrosine hydroxylase (TH) expression and dopamine content in the striatum of 2VO rats. Furthermore, expression levels of SP in the striatum and laryngopharyngeal region were evaluated.

## 2. Results

### 2.1. Physiological Characteristic Parameters and Cerebral Blood Flow

**Figure 1:**
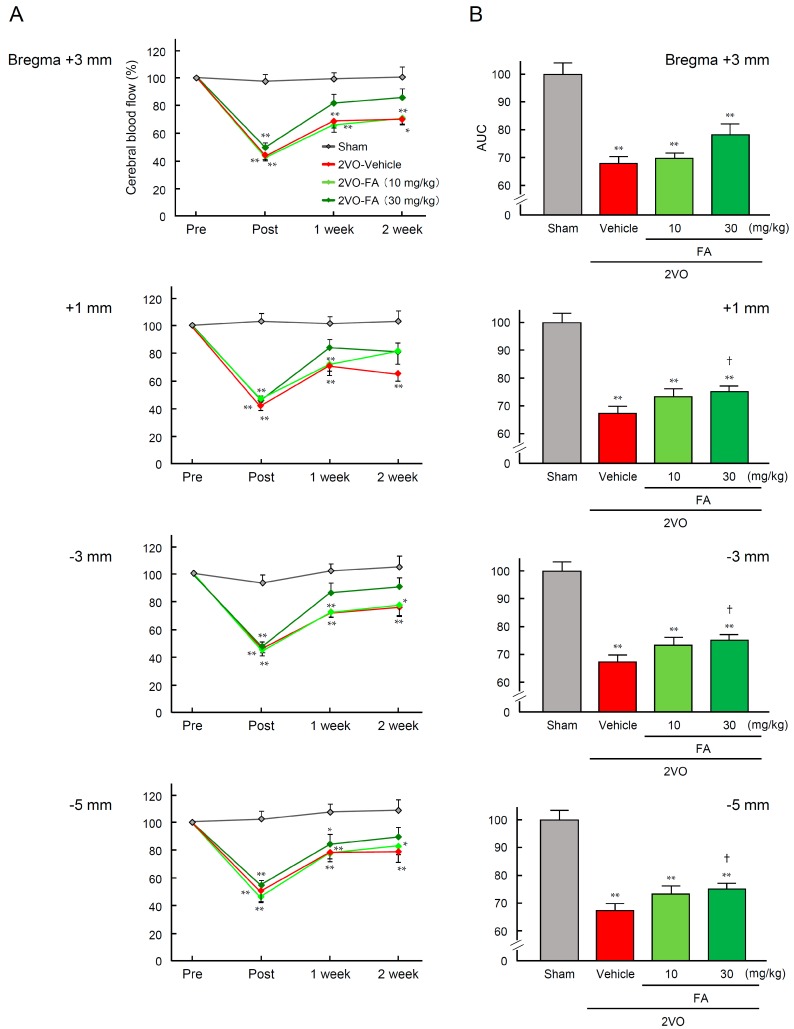
Effects of chronic treatment with ferulic acid (FA) on temporal change in cerebral blood flow (CBF) in 2VO rats. Surficial blood flow at four rostrocaudal levels in the cortex (+3.0, +1.0, −3.0, and −5.0 mm rostrocaudal and 3.0 mm lateral from bregma) was sequentially measured before (Pre) and immediately, 7 and 14 days after (Post) 2VO using laser Doppler flowmetry (**A**); Values of CBF are represented by the means of both hemispheres’ measurements. The area under the curve (AUC) for CBF was determined in each group (**B**). The data are represented as means ± S.E.M.; *n* = 6–9 in each group. *, ** *p* < 0.05, 0.01 compared with the sham-operated vehicle group. ^†^ *p* < 0.05 compared with the 2VO-vehicle group.

2VO caused body weight loss and a decrease in survival rates in the rats (Table 1). Treatment with FA did not affect these parameters 14 days after 2VO. In the 2VO-vehicle group, the values of cerebral blood flow (CBF) in the surficial cortex at four rostrocaudal levels were significantly decreased to an average of 46.8% of the baseline immediately after 2VO, and gradually restored to 71.8% over the 14-day duration of the experimental period (Figure 1A). A similar pattern of change in CBF values was observed in the 2VO-FA groups, although the recovery in CBF after 2VO tended to be enhanced. The higher dose of FA slightly, but significantly, increased the area under the curve (AUC) of CBF only in the parietal cortex (−3 mm caudal from bregma) after 14 days post-2VO (Figure 1B).

### 2.2. Ferulic Acid (FA) Suppresses Ligation of Bilateral Common Carotid Arteries (2VO)-Induced Swallowing Dysfunction

**Figure 2:**
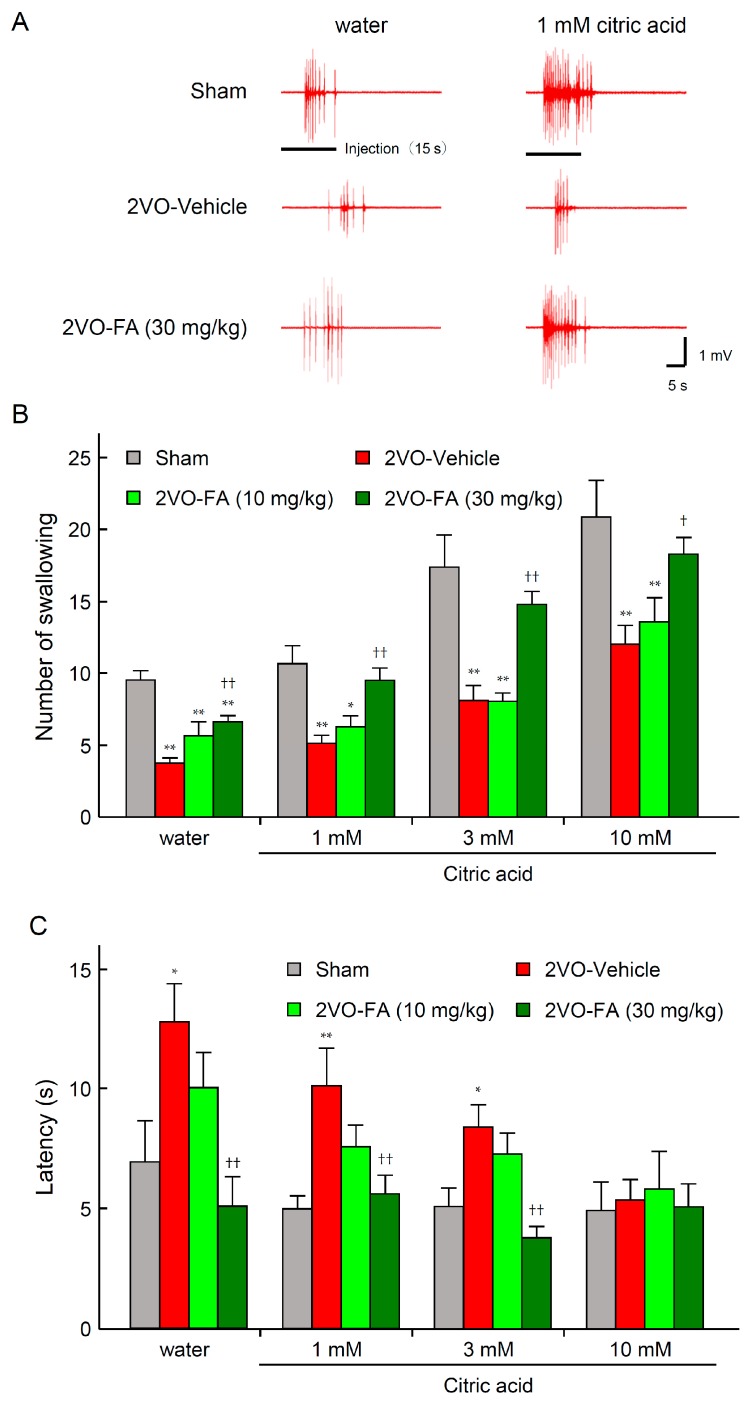
Effects of chronic treatment with FA on swallowing reflex in 2VO rats. Typical mylohyoid Electromyogram (EMG) activity with swallowing reflex elicited by distilled water or citric acid (1–10 mM) in each group at 14 days post-2VO (**A**); Effects of FA on the mean number of swallowing events (**B**) and latency for the first swallowing response (**C**) in each group (*n* = 6–9). The data are represented as means ± S.E.M. *, ** *p* < 0.05, 0.01 compared with the sham-operated vehicle group. ^†^, ^††^ *p* < 0.05, 0.01 compared with the 2VO-vehicle group.

The effects of chronic treatment with FA on swallowing reflex in 2VO rats are shown in Figure 2. Electromyogram (EMG) activities of mylohyoid muscle recorded from representative rats from each group at 14 days post-2VO or sham-operated are shown in Figure 2A. A stimulus by 50 µL distilled water delivered to the laryngopharyngeal region successfully evoked nine swallowing responses during 15 s of infusion, and subsequently, 30 s in the sham-vehicle rat. Approximately 20 swallows with a higher frequency were evoked when citric acid was used as the stimulating solution instead of distilled water. In the vehicle-treated 2VO rats, swallowing responses induced by distilled water and citric acid were both markedly attenuated. On the other hand, the 2VO-FA (30 mg/kg) rat exhibited an almost intact swallowing reflex against both distilled water and citric acid. Figure 2B quantitatively illustrates that 2VO significantly decreased the mean number of swallowing events elicited by distilled water in the vehicle group, which was largely reversed by chronic treatment with 30 mg/kg FA. The number of swallowing events evoked by the same volume of citric acid solution was remarkably increased and the swallow-enhancing potency of this acid increased in a concentration-dependent manner. The citric acid-evoked swallowing reflex was also impaired by 2VO, which was evidently restored by the administration of 30 mg/kg FA. As for swallowing latency, distilled water and lower doses (1 and 3 mM) of citric acid required significantly longer durations to trigger the first swallowing response in the 2VO-vehicle group than in the sham-vehicle group. The highest dose (10 mM) of citric acid normally triggered swallowing reflex even in the 2VO-vehicle group. Treatment with 30 mg/kg FA prevented this prolongation in swallowing latency due to 2VO (Figure 2C).

### 2.3. FA Ameliorates Oxidative Stress and Apoptotic Cell Death in the Striatum

To assess the effects of FA on 2VO-induced systemic oxidative stress, serum levels of hydroperoxide in each group were measured using the Diacron-reactive oxygen metabolites (d-ROMs) test just before 2VO and 24, 72 h and 14 days post-2VO (Table 2). 2VO significantly increased this parameter at all time points in the vehicle group, which was completely suppressed in the 30 mg/kg FA-treated 2VO group. The ameliorative effect of 10 mg/kg FA emerged only at 14 days after 2VO.

**Figure 3:**
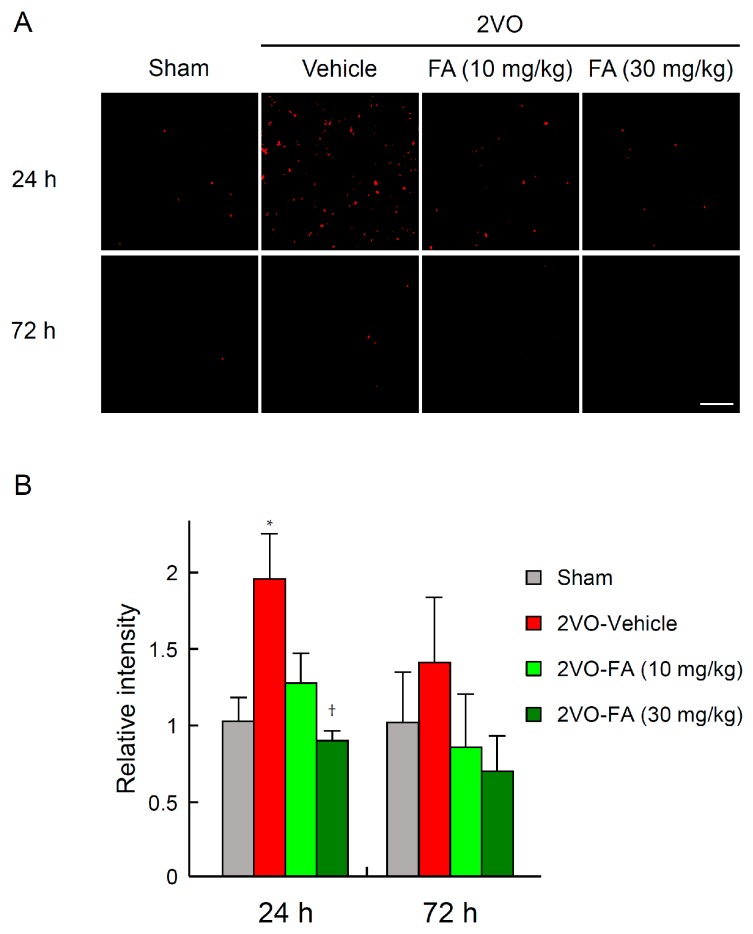
Effects of chronic treatment with FA on striatal oxidative stress after 2VO. Representative results of dihydroethidium (DHE) staining for superoxide production at 24 and 72 h post-2VO in the striatum from rats in each group (**A**), scale bar = 50 µm; Fluorescence intensity of oxidized DHE was quantified using imaging software focused in the relevant areas (**B**). The values of fluorescence intensity of each group are represented as means ± S.E.M. relative to those of the vehicle group; *n* = 4–9. * *p* < 0.05 compared with the sham-vehicle group, ^†^ *p* < 0.05 in comparison to the 2VO-vehicle group.

Intracellular O_2_^−^ generation induced by 2VO in the striatum was detected by histological staining with the fluorescent probe dihydroethidium (DHE) (Figure 3A,B). At 24 h post-2VO, O_2_^–^ generation was remarkably elevated in neuronal cells in the brains of vehicle-treated rats, which was ephemeral and attenuated by 72 h. The augmentation of O_2_^–^ generation was significantly suppressed in the 30 mg/kg FA-treated brains. There was no apparent O_2_^−^ generation in the corresponding brain regions of the sham-operated, vehicle-only group.

**Figure 4:**
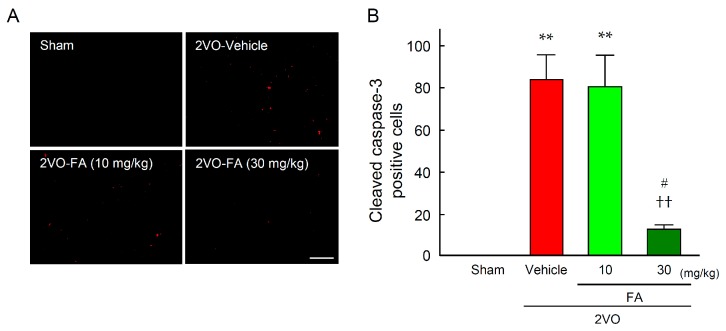
Effects of chronic treatment with FA on apoptotic cell death in the striatum after 2VO. Representative microphotographs of cleaved caspase-3 immunostaining at 14 days post-2VO in the striatum from rats in each group (**A**), scale bar = 100 µm; Quantification of the number of cleaved caspase-3 positive cells was achieved by cell counting in the relevant areas of the rat brains in each group (**B**). Data are represented as means ± S.E.M. from 3–5 rats in each group. ** *p* < 0.01 compared with the sham-vehicle group. ^††^ *p* < 0.05 compared with the 2VO-vehicle group. ^#^ *p* < 0.05 compared with the 2VO-FA (10 mg/kg) group.

To estimate apoptotic cell death, the expression level of cleaved caspase-3 (an activated form of caspase-3) was determined by striatum immunostaining at 14 days post-2VO (Figure 4A,B). The number of cells expressing cleaved caspase-3 was remarkably increased by 14 days after 2VO in the vehicle group. Treatment with 30 mg/kg FA significantly suppressed the overexpression of cleaved caspase-3 in the region.

### 2.4. FA Increases Expression of TH and Dopamine Content in the Striatum

**Figure 5:**
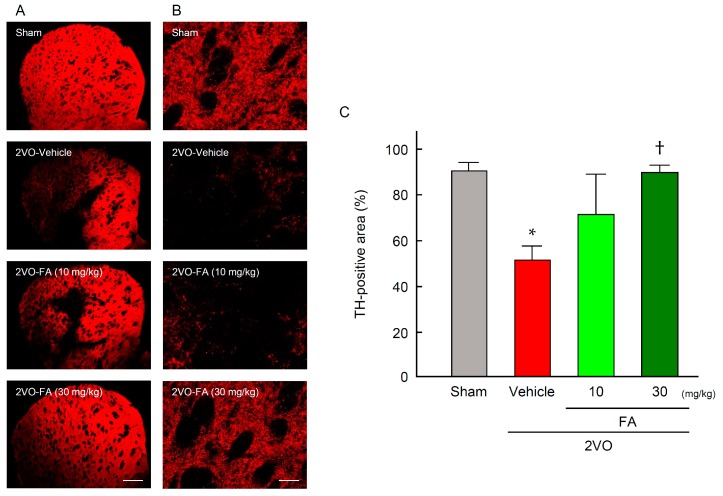
Effects of chronic treatment with FA on expression of tyrosine hydroxylase (TH) in the striatum after 2VO. Representative microphotographs of TH immunostaining at 14 days post-2VO in the striatum from rats in each group at low (**A**) and higher (**B**) magnification. Scale bars: (**A**) 500 µm, (**B**) 50 µm; Quantification of the immunofluorescence was achieved in the relevant areas for rats from each group (**C**). The data are represented as means ± S.E.M. from 3–5 rats in each group. * *p* < 0.05 compared with the sham-vehicle group. ^†^ *p* < 0.05 compared with the 2VO-vehicle group.

**Figure 6:**
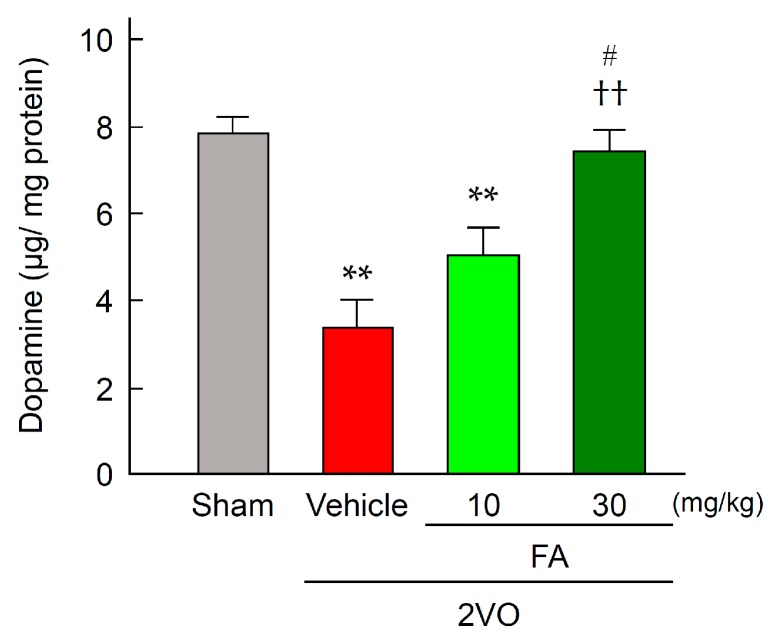
Effects of chronic pretreatment with FA on dopamine content in the striatum after 2VO. Dopamine content was measured by HPLC in the striatum from rats in each group at 14 days post-2VO. Data are represented as means ± S.E.M. from 6–9 rats in each group. ** *p* < 0.01 compared with sham-vehicle animals. **^††^** *p* < 0.01 compared with the 2VO-vehicle group, ^#^ *p* < 0.05 compared with the 2VO-FA (10 mg/kg).

Expression of TH was determined by striatum immunostaining 14 days post-2VO (Figure 5A–C). In the sham-vehicle group, TH immunoreactivity was detected in the nerve endings in the whole area of striatum (Figure 5B). The expression level of TH was remarkably decreased after 2VO in the region of the 2VO-vehicle group. Treatment with 30 mg/kg FA significantly relieved the decrease in expression of this enzyme that was induced by 2VO. In accord with the results from TH immunohistochemistry, a decrease in dopamine content in the striatum of 2VO group was revealed (Figure 6). In contrast, the 2VO-FA (30 mg/kg) exhibited a similar level of dopamine to those observed in the sham-vehicle rats.

### 2.5. FA Maintains SP Expression in the Striatum and Laryngopharyngeal Region

**Figure 7:**
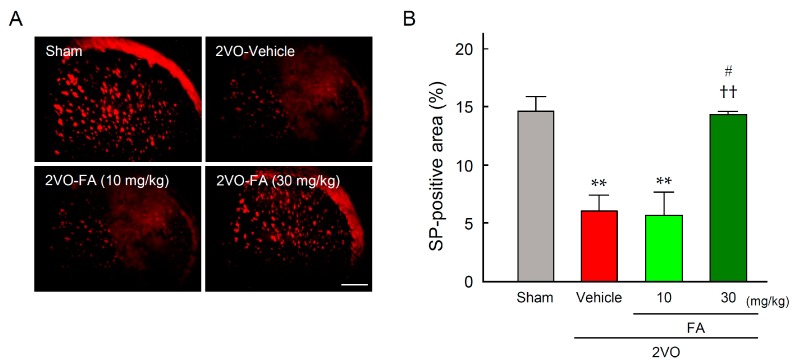
Effects of chronic pretreatment with FA on expression of substance P (SP) in the striatum after 2VO. Representative microphotographs of SP immunostaining at 14 days post-2VO in the striatum of rats from each group (**A**), scale bar = 500 µm; Quantification of the immunofluorescence was achieved in the relevant brain areas from rats in each group (**B**). The data are represented as means ± S.E.M. from 3–5 rats in each group. ** *p* < 0.01 compared with the sham-vehicle group. ^††^ *p* < 0.01 compared with the 2VO-vehicle group. ^#^ *p* < 0.05 compared with the 2VO-FA (10 mg/kg) group.

Expression of SP was determined by immunostaining in the striatum at 14 days after 2VO (Figure 7A,B). The expression level of SP was remarkably decreased by 2VO in the corresponding brain regions of the 2VO-vehicle group. This decrease was significantly alleviated in the 2VO-FA (30 mg/kg) group.

**Figure 8:**
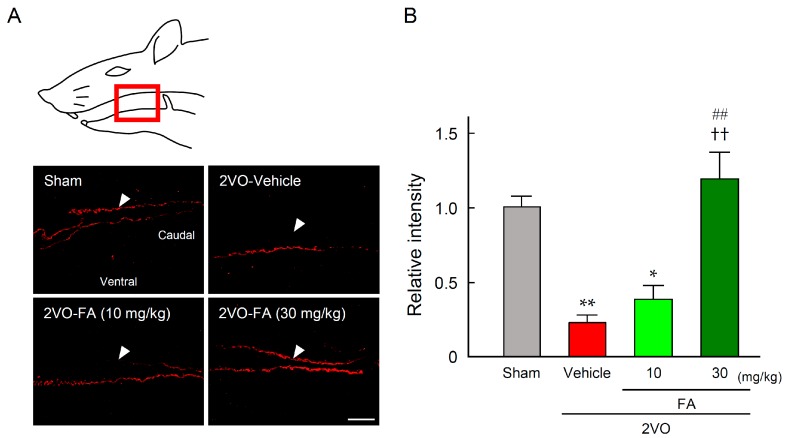
Effects of chronic pretreatment with FA on expression of SP in the laryngopharyngeal region after 2VO. Representative microphotographs of SP immunostaining at 14 days post-2VO in the laryngopharyngeal region (corresponding to the area surrounded by the red frame in the upper illustration) from rats in each group (**A**), arrowheads point out the dorsal mucous membranes, scale bar = 50 µm; Quantification of immunofluorescence was achieved for the relevant regions from rats in each group (**B**). The data are represented as means ± S.E.M. from 3–5 rats in each group. *, ** *p* < 0.05, 0.01 compared with the sham-vehicle group. ^††^ *p* < 0.01 compared with the 2VO-vehicle group. ^##^ *p* < 0.01 compared with the 2VO-FA (10 mg/kg) group.

Figure 8A presents representative photographs of SP immunostaining of the laryngopharyngeal region from each group. In the 2VO-vehicle rats, lower levels of SP expression were observed in the dorsal mucous membrane as compared to the sham-vehicle rats. Treatment with 30 mg/kg FA maintained SP expression in this region. Quantification of expression levels of SP in each group confirmed that 30 mg/kg FA significantly suppressed the depression of SP in 2VO rats (Figure 8B).

## 3. Discussion

In the present study, we demonstrated that chronic oral treatment with FA showed significant protective effects against attenuation of the swallowing reflex in a rat chronic cerebral hypoperfusion model. The higher dose of FA (30 mg/kg) decreased O_2_^−^ production and the number of cleaved caspase-3-positive cells in the striatum, suggesting that FA can protect neuronal cells against 2VO-induced apoptosis by ameliorating ischemic oxidative stress. In addition, we showed that the same dose of FA that revealed the antioxidative and antiapoptotic effects maintained TH expression and dopamine content in the striatum of the rats, which was associated with a certain expression level of SP, a key molecule triggering the swallowing reflex, in the striatum and laryngopharyngeal region. These results suggest that FA ameliorated the swallowing dysfunction by protecting the extrapyramidal dopamine-SP system against the ischemia-induced oxidative damage in the rat brain.

FA has been shown to directly scavenge reactive oxygen species (ROS) [23,24] and activate multiple antioxidant responses including the induction of antioxidative enzymes such as heme oxygenase, superoxide dismutase and catalase, and ameliorate lipid peroxidation [25,26]. A number of studies employing animal models have suggested that chronic administration of FA can prevent and/or relieve neuronal impairment in Alzheimer’s and Parkinson’s diseases as well as cerebrovascular disorders [26]. Oxidative stress has been considered to play a significant role as a predisposing factor in the pathogenesis and progression mechanisms of these debilitating illnesses. Recently, some studies have demonstrated that FA protects against neuronal cell death induced by focal cerebral ischemia in a rodent middle cerebral artery occlusion model [28,30]. FA decreased the infarction volume and suppressed neuronal apoptosis and inflammatory responses caused by focal ischemia following reperfusion. Our present study showed that chronic treatment with FA suppressed the 2VO-induced enhancement of oxidative stress in plasma and O_2_^−^ generation in the rat brain. Previous studies have demonstrated that cerebral hypoperfusion by 2VO results in global oxidative damage through overproduction of ROS, presumably due to mitochondrial dysfunction, and triggers the apoptotic process in neuronal cells [31]. We revealed that FA markedly decreased the number of cells overexpressing activated caspase-3, the executioner of apoptosis, which could be attributed to the improved antioxidant status and reduced oxidative stress in the hypoperfused brain. On the other hand, FA has been reported to exert a vasodilatory effect [32]; however, we observed that FA relieved the 2VO-induced damage without a prominent effect on CBF in rats, suggesting that the cerebroprotective effect of FA can be mainly due to its antioxidant activity.

Sensory inputs evoked by mechanical or chemical stimulation in the laryngopharyngeal area drive the activity of bulbar CPG and evoke reflexive swallowing [4]. In agreement with previous studies [8,33], we confirmed that 2VO prolonged the onset latency to the first swallowing and decreased the numbers of swallows evoked by the stimulation solutions. Zhang et al. [8] revealed that the same rat model has a significant bacterial infection of the lungs two weeks after 2VO, suggesting that this animal model meets the research on cerebral ischemia-related oropharyngeal dysphagia. We observed that in the 2VO-vehicle rats, swallowing responses induced by a small amount of distilled water or lower concentrations (1 or 3 mM) of citric acid were markedly attenuated. These results imply that the sensory inputs chemically evoked by both water and acid in the laryngopharyngeal area may be attenuated. A high concentration (10 mM) of citric acid could evoke normal swallowing reflex responses, supporting the postulation that the activity of CPG remains intact in the 2VO rats in which blood supply to the brainstem is considered to be maintained by bilateral vertebral arteries.

The swallowing reflex evoked by water is believed to depend on the activation of water receptors [34], whereas acid stimulation may depend on transient receptor potential vanilloid type 1 (TRPV1) channels expressed in laryngopharyngeal afferent nerve fibers [35]. Activation of TRPV1 by acidity, heat, or capsaicin is known to induce SP release from laryngopharyngeal afferent nerve endings, triggering the swallowing and cough reflex [36]. Treatment of stroke patients with angiotensin-converting enzyme inhibitor, which can inhibit the degradation of SP, has been reported to increase the serum level of SP and relieve aspiration [37]. To date, several lines of evidence suggest a link between the disturbance in the extrapyramidal nigrostriatal dopaminergic-SP system and oropharyngeal dysphagia in humans and animals. Patients with infarction in the basal ganglia and Parkinson’s disease, who also have dopaminergic dysfunction, frequently present with oropharyngeal dysphagia [9,10]. Therapeutic drugs for treatment of Parkinson’s disease that can increase dopaminergic neurotransmission have been shown to improve swallowing reflex in these patients [13,14]. It has also been demonstrated that nigrostriatal dopaminergic transmission positively regulates the gene expression of SP in the striatum [38] and SP content in laryngopharyngeal mucosa [39]. These findings support a significant role of the dopamine-SP system in regulating the swallowing reflex. The 2VO model reproduces impairment of swallowing reflex in a manner similar to that observed in post-stroke patients with dysfunction of the nigrostriatal dopamine-SP system [8,33]. We confirmed that the expression levels of TH, a rate-limiting enzyme for the synthesis of catechol amines including dopamine, significantly decreased in the striatum at 14 days post-2VO, which was concurrent with a decrease in dopamine content of the region. Furthermore, expression levels of SP in both the stratum and laryngopharyngeal mucosa were lowered, suggesting that chronic cerebral hypoperfusion led to functional perturbation of the dopamine-SP system of 2VO rats.

On the other hand, we found that chronic treatment with FA maintained both dopamine and SP levels in the striatum and improved swallowing reflexes in 2VO rats, indicating that appropriate regulation of the dopamine-SP system by FA contributes to maintaining this reflex. Previously, cilostazol has been reported to prevent aspiration pneumonia through the improvement of the swallowing reflex in post-stroke patients [40]. Besides anti-platelet effects, cilostazol has been demonstrated to exert anti-apoptotic and anti-inflammatory effects [18] and to improve the swallowing function [8], attributed to the activation of the CREB pathway in 2VO rats. FA has also been reported to enhance the phosphorylation of CREB and inhibit the caspase-3-dependent apoptotic pathway [41]; therefore, this mechanism may contribute in part to the anti-apoptotic effects observed in our study. We demonstrated that FA exhibits the above-mentioned beneficial effects at a low dose of 30 mg/kg/day. The efficacy of FA is comparable to that of “designer drugs”, including cilostazol. Taking into account its low toxicity as a dietary supplement, the long-term use of FA can be accommodated for the therapy of chronic complications with fewer adverse effects. Furthermore, FA has been shown to up-regulate a wide variety of cytoprotective systems, including anti-inflammatory effects [26], suggesting its potential for the reduction of sequelae (including dysphagia) and improvement of prognosis in post-stroke patients. Our present study suggests that chronic supplementation with FA can be low-cost and efficacious preventive therapy for stroke. Since strokes are caused suddenly, only a small part of the patients can be received the benefits from the use of alteplase within the narrow therapeutic time window. In consideration of the difficulty in ensuring the prompt and secure treatment for cerebral stroke, an aggressive preventive care should be developed, especially in the high-risk patients with a past history and/or hypertension. Further basic and clinical studies need to be conducted to demonstrate the clinical utility of FA and for the future development of alternative medicines for the prevention and therapy of post-stroke oropharyngeal dysphagia.

## 4. Materials and Methods

### 4.1. Animals and Treatments

One hundred and thirty seven male adult Sprague–Dawley rats (10 weeks old) weighing 330–350 g were purchased from Japan SLC, Inc. (Hamamatsu, Japan) and housed under a temperature- (23 ± 0.5 °C) and humidity- (55% ± 10%) controlled environment with a 12/12-h light–dark cycle. The rats were provided with standard rodent chow (CE-2, CLEA Japan, Inc., Tokyo, Japan) and water ad libitum. After acclimating for 1 week, the rats were divided into the following four groups: Group I was the sham-vehicle group (*n* = 27), orally treated with saline containing 0.5% carboxymethyl cellulose as vehicle and subjected to sham-2VO operation; Group II was the 2VO-vehicle group (*n* = 42), treated with the delivery vehicle and subjected to 2VO; Groups III and IV were 2VO-FA groups (*n* = 32 and 36), which were treated with 10 and 30 mg/kg FA, respectively, and subjected to 2VO. A volume of 0.3 mL/100 g body weight of the solutions was delivered via a stomach tube once daily for 3 weeks (1 week before and 2 weeks after 2VO). All experiments were performed in compliance with the Guiding Principles for the Care and Use of Laboratory Animals approved by the Japanese Pharmacological Society, and the guidelines were approved by the Ethics Committee on Animal Care and Animal Experimentation at the Josai University (approval number H25041, 3 April 2013). The number of animals used was carefully estimated and kept to the minimum necessary for meaningful interpretation of the data. Animal discomfort was also minimized.

### 4.2. 2VO Procedure

Cerebral hypoperfusion was induced by 2VO in rats, as previously described [8,33]. Rats were anesthetized using isoflurane, with 5% for induction and 2% for maintenance. After fixing a rat in the prone position, a small ventral midline incision was made. Then, the bilateral common carotid arteries were exposed and carefully separated from the carotid sheath and vagus nerves. Both arteries were double ligated with a 6-0 silk suture and cut between the ligations. Sham-operated rats were treated with the same surgical procedures without the carotid arterial ligation.

### 4.3. Measurement of CBF

Surficial blood flow at four rostrocaudal levels in the cortex (+3.0, +1.0, −3.0, and −5.0 mm rostrocaudal and 1.5 mm lateral from bregma) was measured using laser Doppler flowmetry (ATBF-LC1, Unique Medical Co., Ltd., Tokyo, Japan). Measurements were performed sequentially over the experimental period; just before, immediately after, 7 days after, and 14 days after 2VO. CBF values were represented by the means of both hemispheres’ measurements.

### 4.4. Measurement of Swallowing

Measurement of swallowing was performed as described previously [42]. After 14 days post-2VO, the rats were anesthetized with urethane (1.0 g/kg, i.p.) and the head was fixed in a stereotactic frame in the supine position. A midline incision was made in the neck, and to remove the influences of salivation on the swallowing responses, the bilateral three greater salivary glands (the parotid, mandibular, and greater sublingual glands) were carefully cauterized at the roots of their ducts. An endotracheal intubation was made for maintaining respiration, and the esophagus was cannulated to export the stimulating solutions after swallowing. A polyethylene guide tube was fixed in the mouth, and then another tube for infusion of the stimulating solution was passed through the guide tube, with the tip placed into the pharynx. A small amount (50 µL) of distilled water or citric acid solution (1, 3, and 10 mM) as the stimulatory solution was applied to the laryngopharyngeal region by an infusion pump (flow rate: 3.3 µL/s, 15 s). Swallowing reflex elicited by infusion of the stimulating solutions was identified by EMG activities, which were recorded through bipolar stainless-steel wire electrodes inserted into the unilateral hyoid muscle, and by visual observation of the laryngeal movement. Mylohyoid EMG activity was amplified (MEG-5100, Nihon Koden, Tokyo, Japan) and stored on a hard disk for later analysis using a data acquisition system (PowerLab/4s, AD Instruments, Castle Hill, Australia). The onset latency to the first swallowing and the number of swallows during infusion of the stimulating solution (15 s), and subsequently 30 s, was counted in each rat.

### 4.5. Measurement of Systemic Oxidative Stress

Temporal changes in systemic oxidative stress of each group were assessed by measuring the serum levels of hydroperoxide just before and 24, 72 h, and 14 days after 2VO using an active oxygen-free radical autoanalyzer (Free Radical Elective Evaluator: F.R.E.E., Grosseto, Italy) and the d-ROMs test kit as previously reported [43]. The results of the d-ROMs test were expressed in arbitrary units called “Carratelli units” (U. CARR), where 1 U. CARR corresponds to 0.08 mg/100 mL H_2_O_2_. F.R.E.E. and the kits were purchased from Diacron International (Grosseto, Italy).

### 4.6. Evaluation of O_2_^−^ Production by DHE Staining

Intracellular O_2_^−^ generation induced by 2VO in the striatum was detected by DHE staining [43]. Coronal brain sections (30-µm thick) were incubated with DHE (10 µmol/L, Sigma-Aldrich, St. Louis, MO, USA) in 10 mM phosphate-buffered saline (PBS, pH 7.4) for 30 min at 37 °C. After washing and mounting the sections on slide glasses, three microscopic fields of the relevant regions were captured and the fluorescence intensity of oxidized DHE in each field was evaluated using an All-in-One fluorescence microscope (BZ-X700, Keyence, Osaka, Japan). The entire histopathological evaluation was performed by blind evaluation without knowledge of the treatment.

### 4.7. Immunohistochemistry

Striatum expression levels of cleaved caspase-3 and TH 14 days post-2VO were analyzed by immunohistochemical staining. SP expression in the stratum and laryngopharyngeal region was also examined. The brain was perfused with cold saline and fixed with PBS (pH 7.4) containing 4% paraformaldehyde. Coronal brain sections (30-µm thick) for the striatum and sagittal sections (20-µm thick) for the laryngopharyngeal region were incubated with goat serum-blocking solution (S-1000, Vector Laboratories, Youngstown, OH, USA) in 0.3% Triton X-100 in PBS (PBST) for 1 h at room temperature. These sections were incubated overnight with anti-cleaved caspase-3 rabbit monoclonal antibody (1:100; #9664, Cell Signaling Technology, Danvers, MA, USA), anti-TH antibody (1:1000; AB152, Merck Millipore, Darmstadt, Germany), or anti-SP antibody (1:6000, 20064, ImmunoStar, Wisconsin, USA), and then rinsed in 0.3% PBST, followed by incubation with goat anti-rabbit IgG (H + L) labelled with Cy3 (diluted 1:100; life technologies, DriveRockville, MD, USA) for 1 h at room temperature. Immunofluorescence was visualized and quantified using fluorescence microscopy and an imaging software system as described above. Regarding TH and SP in the striatum, areas having above-threshold fluorescence intensity under a laser beam at constant intensity were determined as immunopositive and evaluated using the All-in-One fluorescence microscope (BZ-X700, Keyence, Osaka, Japan).

### 4.8. Measurement of Dopamine Concentration

Dopamine in the striatum was determined by the method previously described [44]. At 14 days post-2VO, rats were sacrificed under anesthesia and brains were rapidly removed and cut into 2 mm coronal sections by using a rat brain matrix (World Precision Instruments, Inc., Sarasota, FL, USA). The striatum was dissected out on an ice-cold plate according to the atlas of Paxinos and Watson [45]. Tissue samples were weighed and then homogenized by ultrasonification (UR-20P; Tomy Seiko, Tokyo, Japan) in 0.2 M perchloric acid containing 100 µM EDTA. After resting on ice for 30 min, the homogenates were centrifuged (20,000× *g*, 4 °C, 15 min). The supernatant was adjusted to pH 3 with 1 M sodium acetate and filtered through a 0.45 µm membrane filter (Minisart RC; Sartorius Stedim Biotech, Goettingen, Germany). Dopamine levels in the supernatants were determined using high-performance liquid chromatography with a reverse-phase octadecylsilyl column (Eicompak SC-5ODS; Eicom, Kyoto, Japan) and an electrochemical detector (HTEC-500, Eicom). The column temperature was maintained at 25 °C and the detector potential was set at 750 mV. The mobile phase, which consisted of 0.1 M sodium acetate–citrate acid buffer (pH 3.5), 17% methanol, 190 mg/L sodium 1-octanesulfonate, and 5 mg/L EDTA, was pumped at 0.5 mL/min.

### 4.9. Statistical Analysis

Statistical differences between the various groups were assessed with one-way analysis of variance (ANOVA) followed by post-hoc Tukey’s multiple-comparison test. Comparisons within groups were performed with a paired *t*-test.

## 5. Conclusions

In conclusion, chronic oral treatment with FA prevents impairment of the swallowing reflex induced by surgical 2VO in rats. FA suppressed both systemic and cerebral oxidative stress and maintained TH expression and dopamine content in the striatum of the rats, which was associated with expression levels of SP in the striatum and laryngopharyngeal mucosa. These results suggest that FA ameliorates the impairment of the swallowing reflex by protecting the nigrostriatal dopamine-SP system against the hypoperfusion-induced oxidative damage in the rat brain. Chronic supplementation with FA may be an alternative approach for prevention and therapeutics of cerebral ischemia-induced swallowing dysfunction.

## Figures and Tables

**Table 1:** Weight gain and survival rates of each group at 14 days of ligation of bilateral common carotid arteries (2VO).

Group	Relative Body Weights (%)	Survival Rates (%)
Sham	115.2 ± 1.2	100 (12/12)
2VO-Vehicle	99.2 ± 2.2	72.2 (13/18)
2VO-FA (10 mg/kg)	98.8 ± 1.6	62.5 (10/16)
2VO-FA (30 mg/kg)	99.1 ± 2.6	64.7 (11/17)

Values for body weights in each group at 14 days post-2VO are represented as means ± S.E.M. relative to those before 2VO or sham-operated. Body weights in each group before 2VO or sham operation were equal.

**Table 2:** Effects of FA on systemic oxidative stress in cerebral hypoperfusion rats.

Group	Oxidative Stress (U. CARR)		
	24 h	72 h	14 days
Sham	196.5 ± 23.2	267.3 ± 19.7	229.2 ± 19.3
2VO-Vehicle	483.0 ± 13.1 **	551.3 ± 99.0 *	395.6 ± 25.5 **
2VO-FA (10 mg/kg)	415.5 ± 29.0 **	455.8 ± 48.3	249.3 ± 42.8 ^††^
2VO-FA (30 mg/kg)	283.0 ± 25.5 ^††,##^	248.5 ± 29.8 ^†^	263.1 ± 22.3 ^†^

The results of the d-ROMs test were expressed in arbitrary units called “Carratelli units” (U. CARR), where 1 U. CARR corresponds to 0.08 mg/100 mL H_2_O_2_. F.R.E.E. Oral treatment with FA attenuated the increased oxidative stress induced by 2VO. *n* = 4–10. Values are the mean ± S.E.M. *, ** *p* < 0.05, 0.01, vs. sham, ^†^, ^††^ *p* < 0.05, 0.01 vs. 2VO-Vehicle. ^##^ *p* < 0.01 vs. 2VO-FA (10 mg/kg).

## Abbreviations

AUC	Area under the curve
CBF	Cerebral blood flow
CREB	Cyclic AMP-responsive element binding protein
CPG	Swallowing central pattern generator
DHE	Dihydroethidium
d-ROMs	Diacron-reactive oxygen metabolites
EMG	Electromyogram
FA	Ferulic acid; 4-hydroxy-3-methoxycinnamic acid
ROS	Reactive oxygen species
SP	Substance P
TH	Tyrosine hydroxylase
TRPV1	Transient receptor potential vanilloid type 1
U. CARR	Carratelli units
2VO	Ligation of bilateral common carotid arteries
